# An automated approach to the alignment of compound refractive lenses

**DOI:** 10.1107/S1600577522004039

**Published:** 2022-05-18

**Authors:** Sean Breckling, Bernard Kozioziemski, Leora Dresselhaus-Marais, Arnulfo Gonzalez, Ajanaé Williams, Hugh Simons, Paul Chow, Marylesa Howard

**Affiliations:** a Signal Processing and Applied Mathematics, Nevada National Security Site (NNSS), NV, USA; b Lawrence Livermore National Laboratory, Physics Division, Livermore, CA 94550, USA; c Department of Materials Science and Engineering, Stanford University, Stanford, CA 94305, USA; d Department of Physics, Technical University of Denmark, Fysikvej 311, Kgs Lyngby 2800, Denmark; e HPCAT, X-ray Science Division, Argonne National Laboratory, Argonne, IL 60439, USA

**Keywords:** compound refractive lens, alignment, optimization, beamline optics

## Abstract

Compound refractive lenses (CRLs) are established X-ray focusing optics, and are used to focus the beam or image the sample in many beamlines at X-ray facilities. While CRLs are quite established, the stack of single lens elements affords a very small numerical aperture because of the thick lens profile, making them far more difficult to align than classical optical lenses that obey the thin-lens approximation. This means that the alignment must be very precise and is highly sensitive to changes to the incident beam, often requiring regular readjustments. Some groups circumvent the full realignment procedure by using engineering controls (*e.g.* mounting optics) that sacrifice some of the beam’s focusing precision, *i.e.* spot size, or resolution. While these choices minimize setup time, there are clear disadvantages. This work presents a new automated approach to align CRLs using a simple alignment apparatus that is easy to adapt and install at different types of X-ray experiments or facilities. This approach builds on recent CRL modeling efforts, using an approach based on the Stochastic Nelder–Mead (SNM) simplex method. This method is outlined and its efficacy is demonstrated with numerical simulation that is tested in real experiments conducted at the Advanced Photon Source to confirm its performance with a synchrotron beam. This work provides an opportunity to automate key instrumentation at X-ray facilities.

## Abstract 2

The efficacy of an automatic technique to align compound refractive lenses at beamline facilities is demonstrated. The algorithm is presented in its entirety, along with the results of numerical simulations and an implementation at the Advanced Photon Source at Argonne National Laboratory, USA.

## 1. Introduction

Lens-based X-ray imaging and diffraction provide opportunities to measure materials with resolutions ≤ 100 nm with current technologies. These technologies use either full-field imaging or scanning nano-probes, both of which require X-ray focusing optics. Several X-ray lens options have been developed, including Fresnel zone plates, Kirkpatrick–Baez mirrors and compound refractive lenses (CRLs), which excel for different types of experiments. For imaging applications, CRLs are uniquely versatile, as they have focusing characteristics that can be easily tuned in real time during experiments. CRLs comprise a stack of *n* individual lenslets, where the compounded focusing power from each lens element can be changed to tune the effective focal length of the stack. The versatility of CRLs has made them commonplace in synchrotron X-ray experiments, where they are typically used as upstream beam condensers (Vaughan *et al.*, 2011 ▸; Schroer *et al.*, 2005 ▸) or objective lenses in X-ray microscopes (Lengeler *et al.*, 1999 ▸); however, as thick lenses with small numerical apertures, their accurate alignment is rather challenging. Misalignment of CRLs can increase the width of the focal spot or cause its position to move laterally, resulting in an overall reduction of the quality and intensity of the focal point or image. Corresponding astigmatic aberrations are also generated by the X-ray beam, causing image features to blur directionally as the corresponding focal spot stretches nonuniformly. Together these issues can make the corresponding experiments difficult to interpret.

To align a CRL stack, it must be carefully positioned along the two translation directions (*y* and *z*) and two rotational axes (*r*_*y*_ and *r*_*z*_) that are normal to the axis of propagation of the X-ray beam (*x*), *i.e.* the principal axis of the lens. Optical models to describe CRL alignment (Song *et al.*, 2011 ▸) use wavefront simulations to describe the intensity and beam profile as the beam propagates through a CRL with a pre-defined orientation {*y*, *z*, *r*_*y*_, *r*_*z*_} (positions are typically set by motorized translation/rotation stages). Optimal alignment of CRLs is quite challenging, as the translational and rotational degrees of freedom are coupled, *e.g.* for each position, *y*, the total light transmitted varies as the lens is rotated about the *z*-axis, *r*_*z*_. This condition is specific to CRLs, and arises because of the thickness of the lens, as described fully by Song *et al.* (2011 ▸). Simons *et al.* (2017 ▸) derived an idealized analytical form for the optical transmission function through CRLs that uses ray-optics to express this coupling. For a given CRL orientation, which we define by the alignment vector **p** = (*y*, *z*, *r*_*y*_, *r*_*z*_) in the coordinate system described above, the resulting optical transmission function takes the form of a four-variate Gaussian, which we simplify as 

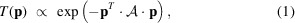

where 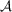 is a symmetric 4 × 4 matrix of terms that mostly depend on the lenslet curvatures and number of lens elements in the CRL. Physically, equation (1) derives from the parabolic shape of each lens element and the Beer–Lambert transmission of X-rays through the lens material.

In practice, CRL alignment is performed in two stages: first, a rough alignment is carried out to ensure that some amount of the X-ray beam transmits through the CRL with coarse motorized scans, or guidance from a reference pinhole on the CRL mount. Second, an X-ray detector (either a camera or intensity monitor) is used to finely align the lens based on the transmitted beam. The fine alignment must be reassessed periodically to account for beam drift, sample quality, or other factors over the course of experiments.

A common strategy for fine alignment is to perform two-dimensional motor scans that measure the transmitted intensity as a function of the CRL’s position and tilt angles by finding the approximate center of the distribution. Accurate sampling of this search space typically requires detailed scans along each independent axis, often necessitating thousands of individual measurements (samplings) to align a single CRL. A faster method is to carry out several cycles of alternating scans between coupled dimensions; however, this still involves hundreds of samples per iteration. For experiments involving frequent CRL alignment [*e.g.* dark-field X-ray microscopy (Kutsal *et al.*, 2019 ▸; Simons *et al.*, 2015 ▸)] or that have many disparate experimental configurations (*e.g.* transmission X-ray microscopy, phase-contrast imaging, or ptychography) the process of aligning, and re-aligning, the CRLs may take a significant fraction of the experimental time.

While experienced researchers may have experiments with minimal drift in the alignment or source, an automated algorithm presents additional benefits to the alignment speed, repeatability, and scalability to multiple simultaneous imaging directions. As X-ray facilities such as synchrotrons and X-ray free-electron lasers (XFELs) often run in continuous operation for weeks or months, a reliable automated method can simplify experimental modifications between users and allow non-expert users to easily realign the experiment if drift or reconfiguration is necessary. This also presents the opportunity in the long term to develop remotely operated or autonomous experimental setups. Automated alignment can also enable facilities to add complexity to their experiments such as multiple probes, smaller incident beam sizes, larger magnifications, or improved spatial resolution depending on the type of experiment. Finally, the repeatability of such an algorithm allows the opportunity to quantify the misalignment and corresponding astigmatism or uncertainty in a measurement – even when substantial jitter or beam-drift is present.

The task of automating the alignment of optics using detection hardware is generally not uncommon, though less common with X-ray lenses at optical facilities. The approach to automating such a task is largely a function of the control hardware, the required accuracy, and the noise levels of the detectors and hardware. One such example is the fine alignment and focus of a camera lens in a wide-field astronomical surveying apparatus. The Robotilter device aligns by optimizing a ‘combo’ score; a positive figure of merit that incorporates both alignment and focus by sampling the four-dimensional search space then performs regression analyses to identify optimal motor positions (Ratzloff *et al.*, 2020 ▸). Fang & Savransky (2016 ▸) demonstrate the alignment of two independent lens assemblies along the same laser beam. Their procedure searches an eight-dimensional parameter space, determining alignment through a fast study of the spatial distribution of light on a near-field camera sensor. For a given position, the resulting image is decomposed into principal components, which provides feedback for an unscented Kalman filter to determine the next position within the search space to evaluate.

In this work we present an alignment method utilizing a variation of the stochastic Nelder–Mead (SNM) online optimization procedure. Nelder–Mead methods, often referred to as simplex methods, are a class of direct search algorithms that iteratively seek to identify parameters that minimize a prescribed real-valued, nonlinear cost function. In our settings, however, we discuss optimization in terms of maximizing a figure of merit (FOM). The classical formulation of Nelder–Mead presumes an unconstrained search space, and a cost function that is strictly convex. These conditions guarantee that a single ‘best’ solution exists, and is unique. If this condition is not met, *e.g.* because of noise, classical Nelder–Mead often fails to locate an optimal solution. In the literature, SNM overcomes these difficulties by mollifying noise through increased sampling, and the occasional use of an adaptive randomized search technique when the path toward an optimal solution is not obvious (Lagarias *et al.*, 1998 ▸). Our implementation slightly deviates from the literature, opting instead to use a procedural local search based on the Sobol sampling technique (Fox, 1986 ▸).

Our description of our method is as follows. We begin in Section 2 by introducing our approach, describing the necessary alignment hardware, and assigning mathematical notation for our methods. In Section 3 we assemble the FOM, which evaluates images from an alignment camera, and returns a transmission estimate akin to (1). We then study this FOM by scanning a 4D space within the limits of motorized stages that position the CRL. These scans are used to provide context for choices made in our algorithm, which is also contained therein. Section 4 reports testing with a series of executions of the algorithm on our apparatus at Advanced Photon Source (APS) (Argonne National Laboratory, USA) as well as several numerical simulations based on data collected there. From the results of collected data, and the numerical simulations, we present a case for improvements and further study of the method in Section 5, and conclude in Section 6.

## 2. Experiment apparatus and mathematical settings

Our apparatus includes the motorized stages that control the position and orientation of the CRL, as well as a simple, self-contained detector assembly (scintillator crystal, imaged onto a camera). We note that the simplicity of the detector and acquisition modes utilized in this work simplifies its implementation in a wide range of experimental settings, allowing versatile and easily customizable configurations. We provide details of our hardware settings and implementation below.

We formulated and tested this method at the Sector 16-ID-D hutch at the APS. This beamline was configured with a double-crystal Si (111) monochromator, and aperture slits to the size of the beam entering the CRL assembly with no additional upstream focusing optics. As such, the beam was nearly collimated and monochromatic with Δ*E*/*E* of 1.0 × 10^−4^. We operated with the monochromator tuned to 9 keV but with the second crystal in the Si (111) monochromator mis-aligned to preferentially remove the third harmonic.

We used a CRL comprising 30 2D Be lenses, each of which has parabolic radii of curvature of *R* = 50 µm and a nominal frame thickness of 2 mm, as purchased from RXOptics GmbH (Lengeler *et al.*, 1999 ▸) with an effective focal length of *f* = 205 mm. The lenses were held in a custom-built V-block and purged with nitrogen to prevent damage via oxidation. The full width at half-maximum (FWHM) of the intensity profile immediately downstream of the CRL stack was 0.30 mm. Assuming ray optics, the 0.30 mm aperture and 60 mm length of the CRL stack dictate that the CRL tilt must be within ±2.5 mrad (0.14°) from the beam axis for some of the collimated incident beam to transmit unobstructed. The CRL assembly itself is mounted on a stage stack, where we make use of four degrees of freedom (two translational and two rotational) in our alignment procedure. We formally denote the translation axes as *y* and *z*, and the rotations about those axes as *r*_*y*_, and *r*_*z*_, respectively; thus, the incident beam axis is defined as *x*. Given that each motor’s position is bounded by travel limits, which are set through software to define the range of the stage’s travel, we denote each limiting interval as 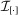 and define our full search space (the set of all permissible CRL orientations) as Ω, the 4D hyper-rectangle of those motor limits is

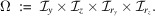

**Figure 1:**
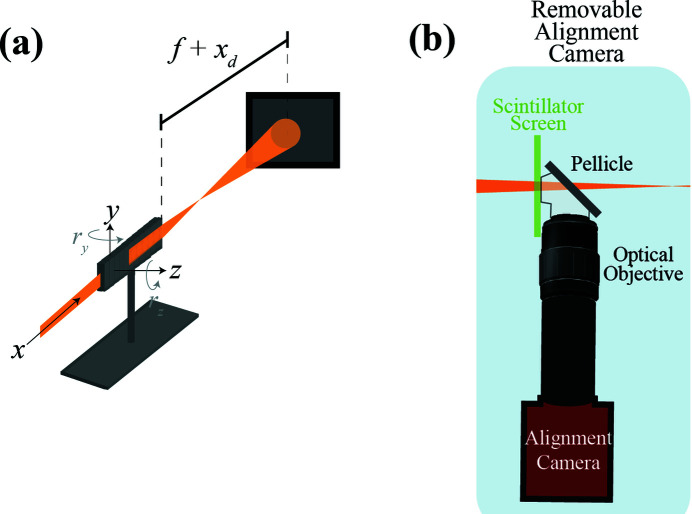
Schematic of the basic layout for the alignment detector, detailed in the blue box labeled Removable Alignment Camera, showing how it fits into a synchrotron experiment. We note that most of the detector optics can be customized based on the availability and beam parameters to set the magnification and field of view for the alignment system.

Alignment images were collected in the near field with a YAG scintillator screen of 10 mm diameter and 50 µm thickness, which was placed 200 mm from the CRL, slightly before the X-ray focus. The YAG scintillator absorbs 65% of the incident X-rays, and a pellicle beam-splitter (ThorLabs BP145B1) was used as a dichroic mirror to separate the X-ray and optical beams. A 7.5× Mitutoyo long-working-distance objective was then used to image the fluorescence from the scintillator crystal onto a Prosilica GC1380 CCD camera. We include a schematic of the apparatus in Fig. 1 ▸.

We note that our implementation used the 2D CCD because this allowed the operator the most flexibility in performing the initial alignment and determining best focus manually. It is important to note that spatially resolved intensity is not required for this algorithm and an energy monitor, such as a photo-diode, could be used in place of the imaging detector, as long as it captures the full transmitted beam.

In our formalism, the beam intensity fluctuates in time, and the exposure time of the camera is user-selected, so we define *t* as the clock time, and Δ*t* as the camera’s exposure time. For a given motor position **p** ∈ Ω, and distance from the camera to the CRL focal plane along the beam axis *x*_*d*_, we express the raw image collected from the near-field CCD camera as *J*_*i*,*j*_(*x*_*d*_, Δ*t*, *t*, **p**), where *i* and *j* represent pixel indices. An upstream ion chamber records a number proportional to the number of photons s^−1^ incident on the CRL, which is integrated over Δ*t*. We denote the recorded incident beam count-rate as ω(Δ*t*, *t*), and use it to normalize the fluctuations inherent to the source from our measured transmission, *i.e.*


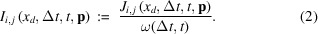

Our intensity measurements do not vary significantly with respect to *x*_*d*_. Additionally, normalizing with the beam energy ω(Δ*t*, *t*) substantially reduces intensity variation in time *t*. Given that we maintain a fixed exposure time Δ*t*, in an effort to aid in readability, we simplify our notation in (2) to *I*_*i*,*j*_(**p**), or *I*_*i*,*j*_ when referring to individual pixels on the normalized image, or simply *I*(**p**) or *I* when discussing the entire image is prudent and convenient.

The initial rough alignment of the CRL to the beam was performed manually. First, the CRLs were removed completely from the beamline and the position of the X-ray beam on the scintillator was recorded. Next, the CRLs were replaced. The CRL is considered aligned to the X-ray beam when the spot centroid after the CRL falls on the same detector location as when the CRL is not used. Additionally, limits to translational and rotational degrees of freedom were determined by locating the edges of the CRL as shadows in the X-ray beam. The algorithm presented below was tested once the beam and CRL positions were located.

## 3. Building and solving the optimization problem

In this section we use the notation from Section 2 to construct an FOM that, when evaluated on our search space Ω, behaves sufficiently convex in the presence of standard synchrotron noise. It is the apparent convexity and noise that motivated our choice of the SNM direct-search algorithm, which is presented in a modified form below in Algorithm A.1[Sec seca.1] in Appendix *A* [App appa].

### 3.1. Camera-based FOM

**Figure 2:**
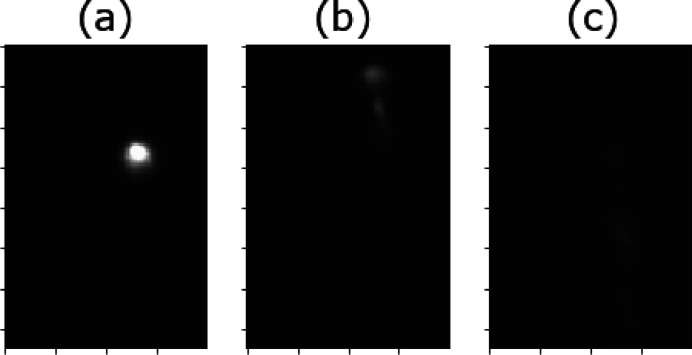
Three examples of positions in the search space with the corresponding conditions for alignment and noise issues. Image (*a*) shows the spot from a well aligned CRL, producing a tight and bright spot, while (*b*) shows a diffuse tail demonstrating its misalignment, and (*c*) shows only a diffuse streak from worse alignment still.

The function we develop below is constructed to mathematically discriminate well alignment from misalignment of the CRL using an imaging sensor placed on the beam. When the CRL is well aligned, we expect to see a small, intense spot at the detector plane. When less well aligned, we should see less intense light spread across a larger region of the camera’s sensor. Examples are presented in Fig. 2 ▸. Here, we develop a robust function that, for a given position **p**, considers the image *I*(**p**) and returns a positive number that quantifies the alignment quality for this system.

For a given motor position **p**, let ξ(*I*) and σ(*I*) denote the median and standard deviation of the resulting detector image *I*(**p**), respectively. With these terms, we identify a region of interest (ROI) within the image as 

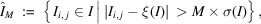

where *M* is a positive, user-selected threshold parameter. We define our FOM as the median value of the pixels within the ROI, *i.e.*


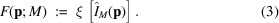

In our apparatus, for any given CRL alignment **p**, the total number of pixels illuminated on the detector plane is substantially lower than half the total number of pixels available. For a well selected threshold *M*, the ROI effectively selects, exclusively, for only those illuminated pixels. Therefore, by selecting the median value of the ROI, our FOM (3) returns a larger number for Fig. 2 ▸(*a*) than either Figs. 2(*b*) or 2(*c*).

To evaluate the behavior of (3), and to ensure that our model appropriately captures the details of the system, we performed samplings of the full 4D search space Ω. While a full, high-resolution 4D scan of Ω would have been ideal, given the time-constaints inherent to the beamline experiments required we limit our scans to: (1) two independent, high-resolution, uniform scans in 2D, and (2) one lower-resolution quasi-uniform scan in 4D.

**Figure 3:**
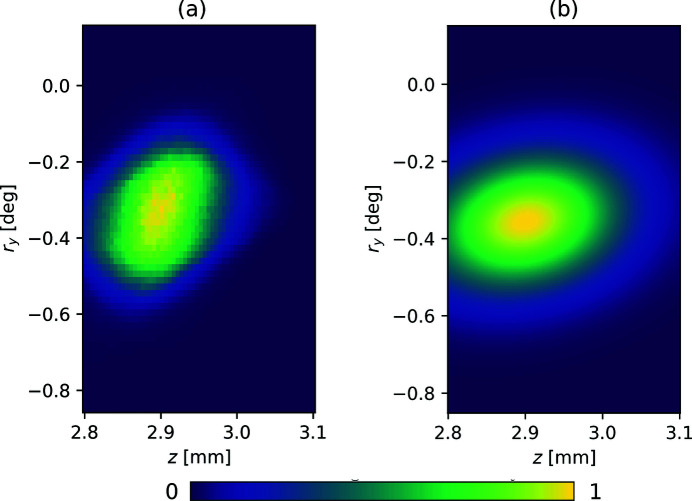
Here we directly compare (*a*) a high-resolution 2D raster scan of the FOM intensity *F*(**p**, *M* = 2) and (*b*) a Gaussian regressions of a lower-resolution 4D raster scan. Both scans depict a feature-scaled scan over (*z*, *r*_*y*_), fixing the remaining variables at 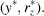

To capture the character of *F*(Ω) in 2D raster scans, we first manually aligned the CRL to establish a ground truth well aligned position, which we shall define as 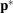 = 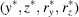 . From this position, we performed two raster scans of the *y* × *r*_*z*_ and *z* × *r*_*y*_ axes, fixing the two off-axis parameters to their optimal coordinates, as defined in 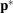 . Using 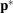 as a central position, we selected sampling intervals for each independent axis. Each interval was then discretized as 60 uniformly distributed positions. We present one of the resulting 2D, 60 × 60-sample raster scan plots in Fig. 3 ▸(*a*).

Collecting a 4D raster scan of *F*(**p**; *M* = 2) at the spatial resolution seen in Fig. 3 ▸(*a*) would require an impractical amount of time. In order to collect as much 4D data during the window of time available, we used a space-filling Sobol sequence of positions in Ω, as implemented in the 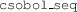 Python package (Fox, 1986 ▸). Given that a Sobol scan does not conform to a grid, we perform a Gaussian regression on the results from which a pixel grid can be evaluated. This regression is implemented in the form 

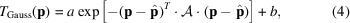

where *a* and *b* are real-valued scalars, 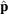 is the position of the peak, and 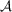 is a symmetric 4 × 4 matrix – a total of 16 variables. One corresponding 2D slice from this regression is depicted in Fig. 3 ▸(*b*).

### 3.2. Optimizing with SNM

In the absence of noise we would formulate the optimization problem in terms of the idealized model (1) such that the optimal solution 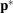 ∈ Ω is given by 

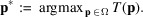

However, since synchrotron experiments are always subject to some type of noise, we constructed our FOM *F*[*I*(**p**)] such that the expectation *E*{*F*[*I*(**p**)]} is a convex function. Based on this assumption, we formulate the problem in stochastic settings such that 

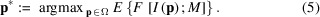

As long as the character of the noise does not cause the FOM to exhibit errant, problematic local maxima, a direct search method is well suited to solve (5).

Nelder–Mead is a direct-search method that is widely used in nonlinear programming problems where derivatives of the associated cost function are not readily available (Nelder & Mead, 1965 ▸; Wright, 1996 ▸). The method works by keeping track of a sample set (or simplex) of *n* + 1 points within the search space, where *n* is the spatial dimension of your search space. The simplex is sorted from ‘best’ to ‘worst’, and proceeds by systematically searching for better positions within the search space. New positions are selected as a function of the spatial orientation of the simplex points, and three real, positive parameters: α, γ, and β, known, respectively, as the reflection, expansion, and contraction values. An implementation of the classic method is packaged within a number of popular scientific computing platforms, namely as 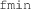 within 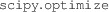 (Virtanen *et al.*, 2020 ▸).

Direct-search methods of this variety are rarely robust to noise. While our FOM (3) is effective at mitigating a substantial degree of the latent measurement noise, it is not entirely eliminated. To account for this, we implemented a modified variety of the SNM method (Chang, 2012 ▸; Li & Zhan, 2014 ▸).

The algorithm we present below deviates from the literature in four major ways. First, we fix the simplex size to 5, since our search space Ω has four dimensions. Second, we formulate our optimization problem to seek a maximal transmission from the detector. The third and fourth modifications both simplify and expedite the FOM sampling criteria seen in the classic versions of SNM, and are detailed below. We enumerate each major step of the SNM procedure with the index *k*, beginning with *k* = 0. For each *k*, when the procedure calls for the FOM to be evaluated, we average *N*_*k*_ evaluations of (3), where 

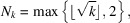

and 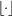 returns the nearest lower integer. For a particular position **p**, sample count *N*_*k*_, and noise-floor threshold *M*, we thus describe the sample-averaged FOM as 

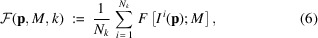

where *I* ^*i*^ is the *i*th image returned from the detector. We determined that the minimum sample of 2 was sufficient, given the noise levels present within the data in our characterization of (3) in Section 3.1. These additional evaluations of (3) come with a minimal time penalty, given that the largest contributor to the wait time per major step *k* is the time required to move the motorized stages.

Our main deviation from the SNM formulation described in the literature is our local Sobol search procedure for situations where simplex contraction fails (*e.g.* the final contingency step in Algorithm A.1[Sec seca.1]). In the non-stochastic formulation of Nelder–Mead, this situation is equivalent to deducing that the simplex spans a spatial region too large to effectively continue improving. There, the algorithm performs a ‘shrinkage’ step, which selectively shrinks the region while keeping the ‘best’ positions. In the stochastic formulations seen in the literature, in lieu of shrinking the simplex, a randomized local or global search is performed (Chang, 2012 ▸; Li & Zhan, 2014 ▸). In these instances, positions within the search space are sampled at random until a new position is found that improves upon the current simplex. In such situations, these SNM formulations utilize an adaptive random search procedure, which potentially performs a global random search of Ω. Our proposed alternative to the randomized search procedure is presented in Algorithm A.2[Sec seca.2].

This deviation was necessary for three reasons. First, while a global random search is a prudent technique to eventually guarantee mathematical convergence of the SNM procedure on paper, in our use case it comes with a potentially un­acceptable time penalty. We first note that the global limits of the motorized stages are substantially larger than the aspect ratio depicted in Fig. 3 ▸, thus a full 4D scan is very unlikely to yield successful improvements. Second, note that we have a strong understanding of the character of our FOM *F*(**p**, *M*): a solitary, convex peak that is sufficiently wide in four dimensions. It is unlikely that this FOM would present errant peaks on a similarly configured apparatus. Finally, after a rough alignment of the optics, enough light is present on the detector to initialize the algorithm. The net consequence is that, in these settings, SNM is unlikely to stray far from a very direct path to an acceptable alignment, precluding the necessity of a global search or highly randomized search.

The final step of any successful execution of an optimization algorithm is the stopping condition. We do not formally specify such a condition in our presentation below, but good choices in our use case could be based on the diameter or volume of the current simplex, the total variation of the FOM on the current simplex, by utilizing a measurement from a far-field detector, or by simply enforcing a maximal iteration tally.

### 3.3. Local Sobol search

Sobol sequences are a low-discrepancy, space-filling sampling technique (Sobol, 1967 ▸). When each point in a low-discrepancy, space-filling sampling is enumerated, and ordered sufficiently well, we find it to be an efficient method to search a bounded region. In our implementations presented below, we find our Sobol sequences using the 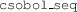 Python package (Fox, 1986 ▸).

To effectively perform a local Sobol sampling during an execution of Algorithm A.1[Sec seca.1], we need to first identify a suitably local region to explore. Given the current motor position **p** we simply select a rectangular region containing **p** determined by two parameters: *k*, the current SNM iteration index, and ε a positive (small) cooling parameter. Our 4D rectangular region 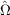 is then determined to be the region between the two following corner positions, 

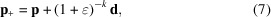




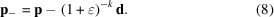

Some care is required when selecting **d** and ɛ. If **d** and ɛ are poorly selected, users run the risk of selecting regions too large or too small to search effectively. We found in our implementation that **d** = *d*〈1.0,1.0,1.0,1.0〉, where *d* is the longest diameter of the initial simplex to be an effective choice, when ɛ is selected such that 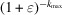 ≃ 1/10, where *k*_max_ is the largest index expected to occur.

When Algorithm A.2[Sec seca.2] is called from within Algorithm A.1[Sec seca.1], the local search space 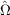 is immediately established by (7) and (8). We then identify an *N*-point Sobol sampling of 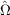 , which we denote as 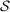 = 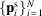 . With the motor positions **p**_*i*_ established, an expeditious route visiting each position is identified using the nearest-neighbor method, discussed further below. Selecting *N* too large creates a situation where the local Sobol search might take a long time to arrive at a region within 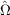 containing an improvement to SNM simplex. Select *N* too small, and the Sobol procedure would likely need to be repeated several times, also causing unnecessary delays. For our implementation discussed below, we found choices of *N* varying between 10 and 50 to be an effective range.

Remark 3.1 A Python implementation and demonstration of Algorithm A.2[Sec seca.2] can be found online (Breckling, 2022 ▸).

Practical implementations of Algorithm A.2[Sec seca.2] require an efficient way to visit each point in the sample space, given the cumulative time required to move the stepper motors. Let 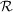 denote a particular course visiting each position within the finite list of coordinates {**p**_1_, **p**_2_,…**p**_*N*_} = 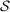 . The time-cost required to move from any one position **p**_*i*_ to another **p**_*j*_ can be estimated by the Euclidean distance |χ_*i*_ − χ_*j*_|. The optimal route 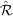 through 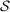 minimizes the total Euclidean distance required to travel to each position. While optimal solutions to this variant of the ‘traveling salesman problem’ cannot be practically identified, a nearest-neighbor solution can, and sufficiently approximates 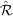 for our use case.

## 4. Implementation results

In this section we present the results of a study to gauge the efficacy of our automated alignment procedure. In Section 4.1 we highlight the results of a single successful alignment outcome. In Section 4.2 we discuss a collection of results, wherein we attempt to quantify the repeatability of Algorithm A.1[Sec seca.1].

### 4.1. Results of a single alignment procedure

In this section we take a qualitative look at the results of a single execution of Algorithm A.1[Sec seca.1]. Upon manually finding a position **p**_0_ such that light is visible on the near-field sensor, we randomly generate an initial five-position simplex χ_0_. This is done by generating random four-vectors, 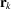 = 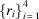 , where each term is sampled from the a uniform distribution on the interval [−0.05, 0.05] (in mm or °, where appropriate), then adding such that 

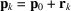

for *k* = 1,…, 4. The sampling interval was selected *ad hoc*, but was large enough to see a substantial perturbation of light present on the near-field camera in each of the four dimensions.

The initial simplex χ_0_ is then used to initialize Algorithm A.1[Sec seca.1]. During initialization, the FOM (6) is evaluated for each position within the initial simplex. We select the Sobol sampling count *N* to be 10, the cooling parameter ɛ = 2.0 × 10^−2^, and the local search region to be determined by the vector **d** = 2.5 × 10^−2^〈1.0,1.0,1.0,1.0〉 (in mm or °, where appropriate).

**Figure 4:**
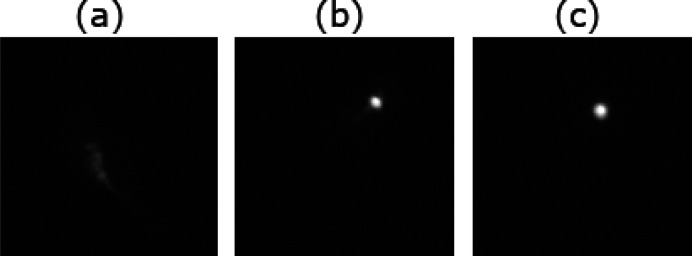
Panels (*a*), (*b*), and (*c*) depict *I*_0_, *I*_6_, and *I*_62_, respectively, which correspond to **p**_0_, **p**_6_, and **p**_62_, the initial, sixth, and final ‘best’ position recorded during one execution of Algorithm A.1[Sec seca.1]. Evaluating each image with the FOM (3), we see that 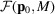 and 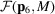 correspond, respectively, to 14.03% and 48.29% of 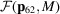 when *M* = 2.0.

Upon initializing Algorithm A.1[Sec seca.1], each position in χ_0_ is evaluated and sorted. The initial ‘best’ position is denoted as **p**_0_, with the corresponding near-field camera image *I*_0_. As the algorithm proceeded to ascend the FOM, we recorded each step, and present the sixth and final ‘best’ positions as **p**_6_ and **p**_62_ with images *I*_6_ and *I*_62_, respectively, in Fig. 4 ▸.

**Figure 5:**
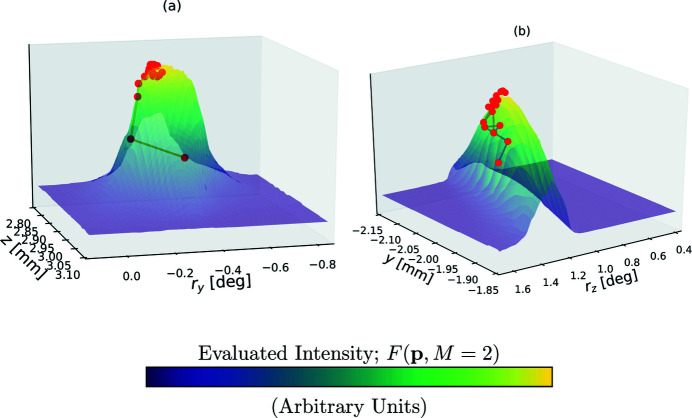
We present the route taken by Algorithm A.1[Sec seca.1] ascending the FOM (6). We overlaid the 16 sequential ‘best’ positions upon a surface plot of the raster scans. Panel (*a*) depicts the *z* and *r*_*y*_ axes, and the *y* and *r*_*z*_ axes can be seen in panel (*b*). The opacity of the surface was decreased so the full paths remain visible.

This execution ceased to present any apparent improvements upon arriving at the position **p**_62_. A signal to end the procedure was then sent. This particular ascent taken by Algorithm A.1[Sec seca.1] was, with rare exception, a direct one. From initialization to quit, the full procedure required only 62 evaluations of 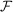 . The incremental improvements seen at most steps appear to be conservatively small. Of those 62 evaluations, there were 16 ‘new bests’, which are presented in Fig. 5 ▸.

### 4.2. A repeatability study

Given that our proposed alignment procedure is inherently stochastic, we performed a Monte Carlo study to evaluate its reproduceability, initializing and executing Algorithm A.1[Sec seca.1] to a satisfactory alignment 30 times. Our goal was to quantify the spread of the final ‘best’ motor positions. Each execution was initialized in the same way as was done in Section 4.1, *i.e.* given a fixed motor position resulting from a rough initial alignment **p**_0_, complete the initial simplex χ_0_ by sampling randomly within the same fixed rectangular region about **p**_0_. In each case, the execution was halted when the resulting spot visible on the alignment camera was sufficiently symmetric and intense.

Over the 30 executions of Algorithm A.1[Sec seca.1], the average tally of evaluations of the sampling-averaged FOM 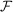 is 63.67, with a median tally of 62, and standard deviation of 4.84 evaluations. This tally is not to be confused with the index *k*, which enumerates the steps within Algorithm A.1[Sec seca.1]. The total number of evaluations of 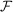 varies from step to step, depending on the location of the simplex. The total-evaluations tally therefore better represents the total time required to fully execute the procedure.

**Figure 6:**
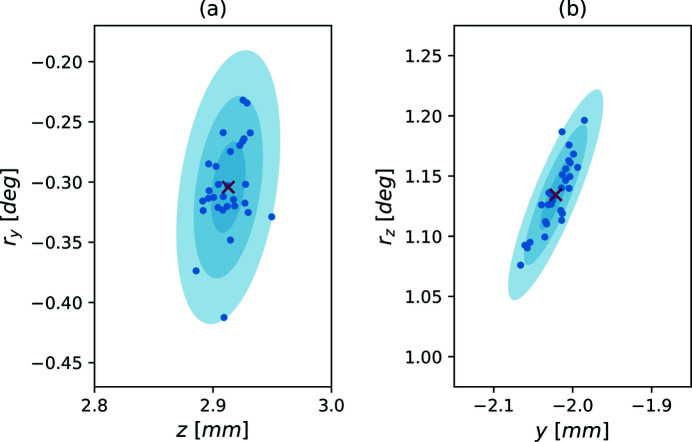
Together, these scatter plots depict the best motor position recorded prior to manual termination of 30 executions of Algorithm A.1[Sec seca.1] (blue dots). The cross (red) represents the average of these recorded positions. We further illustrate the 68, 95, and 99.7% uncertainty regions as concentric ellipses centered at the mean. Panel (*a*) presents the *z* and *r*_*y*_ axes, while the *y* and *r*_*z*_ axes are in given in panel (*b*). Each execution was initialized from the same region in Ω, but the initial simplex was selected randomly (distributed uniformly) about **p**_0_.

Upon sending the stopping command to end the execution, the final ‘best’ position was recorded. Scatter plots of these ‘best’ results are seen in Fig. 6 ▸. We used the spatial statistics of the ‘best’ positions recorded in 30 executions of Algorithm A.1[Sec seca.1] to generate the two-dimensional projections of the 4D elliptical uncertainty regions presented in Fig. 6 ▸. We found that, when the algorithm is manually terminated, 95% of the ‘best’ transmission measurement results were within 90% of the ground truth intensity.

### 4.3. Simulated repeatability study

The principal goals of our simulated studies are to provide validation for the results presented in the previous section. Second, we intend to demonstrate the efficacy of Algorithm A.1[Sec seca.1] in terms of noise. We begin by developing a mathematical model of the FOM (3), accounting for estimates of beam jitter and a paramaterizable additive noise term. We then test the performance of Algorithm A.1[Sec seca.1] using the same settings, while prescribing varying amounts of additive noise.

The modeled FOM 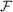 _Gauss_ (9) utilizes the 4D Gaussian fit, *T*_Gauss_ (4), and includes a normally distributed additive noise term, 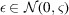 , where ς is a user-selected parameter. A baseline estimate of ς is computed as 5.12 × 10^−3^. This was accomplished by selecting a region of Ω far away from the positions corresponding to well alignment. Additionally, given a beam divergence of 6.5 × 10^−3^ rad, we consider the possibility of random pointing jitter in the beam itself. Thus, we include normally distributed random perturbations on the *r*_*y*_ and *r*_*z*_ axes with a standard deviation of 10% of the beam divergence.

For each Monte Carlo study, we execute Algorithm A.1[Sec seca.1] a total of 30 times, using the model FOM,

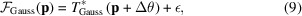

where 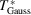 is unit normalized from *T*_Gauss_. Each initial simplex is generated around a fixed position **p**_0_, then populated by selecting nearby positions at random, as was done in the beamline study in Section 4.2. The number of Sobol sampling positions *N* remains set to 10, as with the cooling parameter ɛ = 2.0 × 10^−2^.

Unlike the synchrotron implementation, which was manually exited by the user, an automatic stopping condition within Algorithm A.1[Sec seca.1] is required. In the synchrotron study, on average, 63.67 evaluations of (6) were required. In an effort to make a fair comparison, we forced the automated method to exit upon reaching 64 evaluations.

**Figure 7:**
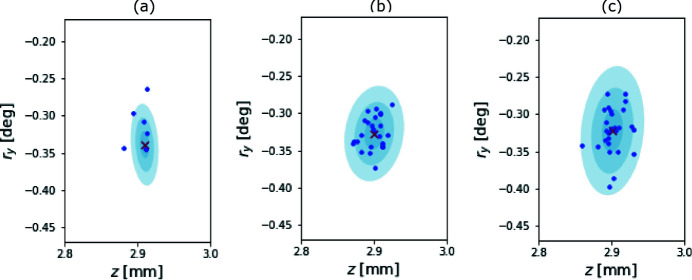
Depicted are the spatial results of three Monte Carlo simulations, in two of the four total dimensions. We varied the additive noise parameter ς between each sampling. Panel (*a*) depicts the case where ς = 5.0 × 10^−4^, (*b*) shows the case where ς = 5.0 × 10^−3^, and (*c*) shows the ς = 5.0 × 10^−2^ case. All results are depicted as blue scatter points above three blue confidence regions. The mean result is presented as a red cross.

We performed three Monte Carlo experiments, each fixing the value for the noise parameter ς to be 5.0 × 10^−*n*^, for *n* = 2, 3, and 4. The results are presented as scatter plots of the final ‘best’ alignment position in Fig. 7 ▸. We observe that, for noise levels comparable with those seen at APS (ς = 5.0 × 10^−3^), the spatial distribution of alignment results of the numerical Monte Carlo simulation qualitatively agree with those seen at the synchrotron Monte Carlo. As expected, we also see that the corresponding confidence regions appear to grow proportionally with ς.

## 5. Discussion and outlooks

**Figure 8:**
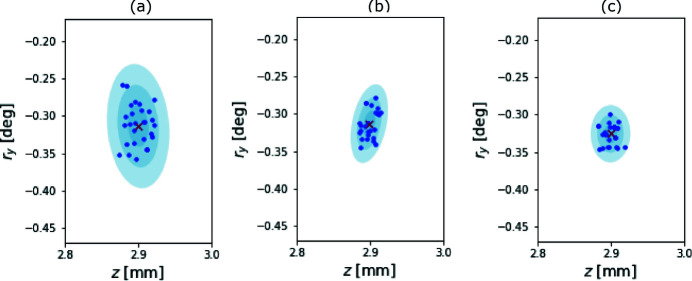
Depicted are the spatial results of three Monte Carlo simulations, in two of the four total dimensions. We included the simulated beam jitter, and fix the additive noise parameter ς = 5.0 × 10^−3^, varying the reflection parameter within Algorithm A.1[Sec seca.1]. Panel (*a*) shows our default choice, α = 1.0, panels (*b*) and (*c*) decrease the parameter to α = 0.75 and 0.5, respectively. The results are depicted as blue scatter points above three confidence regions, and a red cross depicting the mean result.

Further exploration of this technique will likely lead to improvements in speed, accuracy, and precision. In particular, we look forward to identifying reliable choices of the parameters required to initialize Algorithm A.1[Sec seca.1]. For example, in the settings established for the simulated study in Section 4.3, reducing the reflection parameter to α = 0.75, or further to α = 0.5, in Algorithm A.1[Sec seca.1] results in substantial improvements to the spatial statistics. These results are seen in Fig. 8 ▸. Whether or not these changes improve performance on a beamline is an open question.

Second, since an attractive application of this technique is a reliable unsupervised re-alignment procedure, an investigation into automated stopping conditions is required. We anticipate any such condition to be determined by three conditions: a prescribed maximum tally of motor motions, a processing of image data from near or far-field camera sensors, and a time-series study of all evaluations of the FOM.

A robust sensitivity study of the input parameters of Algorithm A.1[Sec seca.1] on the modeled FOM (9) is unlikely to identify optimal parameter settings in real experimental configurations. However, we suspect that efficient and effective parameter selections can be identified with moderate calibration time.

## 6. Conclusions

In this paper we presented a technique to automate the alignment of CRLs. The technique was implemented and tested at the Advanced Photon Source at Argonne National Laboratory. The complete algorithm was presented, along with a study of its efficacy in synchrotron applications using nominal settings. Additionally, we verify and reproduce the experimental tests in a simulated repeatability study. Lastly, we propose a number of improvements which can be verified with more time at an X-ray light source.

## Appendix A

Full details of the algorithms mentioned in the paper are discussed herein, and are enumerated in the order they were introduced above. We again note that examples of the following methods have been provided online (Breckling, 2022 ▸).

### A.1. Algorithm A.1. Stochastic Nelder–Mead for CRL alignment

Each major step of the SNM method is indexed by *k*, and considers a simplex of positions χ_*k*_ = {**p**_1,*k*_,…, **p**_5,*k*_}. The reflection, expansion, and contraction parameters α, γ, and β are required; nominal values are often assigned to be α = 1, γ = 2, and β = 1/2. We additionally require the diameters of the local 4D region **d**, and the cooling parameter ɛ.

*Step 0*. Determine a valid initial simplex of five, non co-linear positions; *i.e.* no more than two points within the simplex should fall on the same straight line in Ω. Additionally, ensure light passing through the CRL is present on at least one of the positions in the initial simplex χ_0_, though preferably all positions. Lastly, evaluate all points in χ_0_ using (6).

*Step 1*. Sort the current simplex χ_*k*_ from ‘best’ to ‘worst’. Denote the best and worst as  and  , respectively.

**If** the stopping criteria is satisfied, **then** exit the procedure.

**Else** let  denote the second lowest-ranked position, then

 
**If** the number of points in χ_*k*_ exceeds 4, **then** remove  from χ_*k*_.

 
**Else** proceed to *Step 2*.

*Step 2*. Compute a centroid  of all remaining points in χ_*k*_. Generate a new point  by reflecting  through  such that

Evaluate

**If**  , **then** add  to the simplex; *i.e.*

and return to *Step 1*.

**Else-If**  , **then** expand the reflection point as

Evaluate  then

 
**If**  , **then** add  to the simplex; *i.e.*

and return to *Step 1*.

 
**Else** add  to the simplex; *i.e.*

 then return to *Step 1*.

**Else-If**  , **then** we compute an outside contraction point, *i.e.*

then evaluate

 
**If**  , **then** add  to the simplex; *i.e.*

then return to *Step 1*.

 
**Else** perform the local Sobol search (Algorithm A.2) to find  and update the simplex

 then return to *Step 1*.

**Else**  , **then** we compute an internal contraction point, *i.e.*

and evaluate

 
**If**  , **then** add  to the simplex; *i.e.*

then return to *Step 1*.

 
**Else** perform the local Sobol search (Algorithm A.2) to find  and update the simplex

 then return to *Step 1*.

### A.2. Algorithm A.2. Local Sobol search

Given a maximal sample count  , the current SNM iteration *k*, a given cooling parameter ɛ, the initial box-width estimate **d**, the simplex χ, and known evaluations of  for all **p**_*i*_ ∈ χ, we identify a local region  and perform space-filling sample.

*Step 1*. Generate the local search region by creating the box  around the current motor position **p** by identifying the corners (7), and (8). Create an *N*-point Sobol sampling of  , denoted as  =  .

*Step 2*. From the current position of all four stepper motors, compute an approximate shortest path through each untested point of  using the nearest-neighbor method.

*Step 3*. Begin evaluating each point within the Sobol sampling sequentially, according to the ordering established in *Step 2*, using the sample-averaged FOM (6)

**If** at any point  , **then** return  *.*

**Else** proceed to *Step 4*.

*Step 4*. **If** no position within S improves the simplex χ, **then** extend the Sobol sample by *N* additional points, and return to *Step 2*.

### A.3. Algorithm A.3. Nearest neighbor

Given a list of spatial coordinates  in  :

*Step 0*. Denote the nearest-neighbor solution as the set  , and initialize as the empty set ∅. Given the initial position of the motors **p**_0_, determine the nearest position within  to **p**_0_, and denote that point as  .

*Step 1*. Remove  from the set  Let *m* denote the number of positions contained in  . Enumerate  as **p**_*m*+1_, and add it to

*Step 2*. If any positions remain in  determine the nearest position in  to **p**_*m*+1_. Denote that position as  , and return to *Step 1*.
